# The Role of Macrolides in Noncystic Fibrosis Bronchiectasis

**DOI:** 10.1155/2011/751982

**Published:** 2011-09-05

**Authors:** Bruna de Campos Guimarães e Figueiredo, Cássio da Cunha Ibiapina

**Affiliations:** Department of Pediatrics, Federal University of Minas Gerais, Brazil

## Abstract

*Objective*. The present study aims at reviewing the main publications on the use of macrolides as immunomodulators in patients with noncystic fibrosis bronchiectasis. *Source of Data*. The Medline database was our source of data for this research carried out until June 2011, using the key words: macrolides and bronchiectasis, while searching for original articles and reviews. *Summary of Data*. Seven clinical studies that evaluated the action of the macrolides in patients with bronchiectasis were found. There was the sputum volume, reduction in pulmonary exacerbation frequency, and in the use of antimicrobial treatment, in addition to pulmonary function improvement. *Conclusions*. Anti-inflammatory action and immunomodulatory effects can be attributed to macrolides when administered in low doses and on the long term. This use has been well established both in diffuse panbronchiolitis and in cystic fibrosis. Evidence indicates possible benefits in bronchiectasis. Future studies are needed, though, to establish the ideal dose and treatment duration and to understand the implications in the generation of microbial resistance.

*“When patients have bacteria that are resistant to all antibiotics, prescribe erythromycin, leave them on it for a long time, and they will do much better”*

Dr. Harry Shwachman, 1950

## 1. Introduction

Macrolides have been known for their antimicrobial actions since 1952 [1]. Their mechanism of action consists of inhibiting protein synthesis by linking to the 50S ribosomal subunit of susceptible microorganisms [2]. Macrolides are widely used to treat infections of soft tissues and of the respiratory tract due to their efficacy against Gram-negative and Gram-positive bacteria, including intracellular germs such as Chlamydia and Legionella [3–5]. 

They are considered safe and easily tolerable. Their main side effects are nausea, vomiting, diarrhea, and abdominal pain, which become more evident when erythromycin is used in place of the other macrolides [6]. Transitory alteration of transaminases and association with ventricular arrhythmias are typically rare [2].

Mounting evidence suggests that macrolide antibiotics have both anti-inflammatory and immune-modulatory properties and are thus beneficial to chronic pulmonary diseases such as diffuse panbronchiolitis, cystic fibrosis, asthma, and bronchiectasis. These properties were suspected upon the realization that erythromycin decreased the need for corticosteroids in asthma treatment [7].

It must be pointed out that immunemodulation is the suppression of inflammation and immune hyperactivation without causing immune depression (immunsuppression) [8]. 

The anti-inflammatory effects of macrolides were initially proved on patients with diffuse panbronchiolitis (DPB) [9–15], a chronic inflammatory disease of the respiratory bronchioles of undetermined cause, affecting mostly Asians. The main symptoms are chronic cough, purulent sputum, dyspnoea, and sinobronchial syndrome. It is commonly associated with chronic infection by *Pseudomonas aeruginosa* and, if not treated, leads to high mortality rates due to respiratory failure.

Historically, DPB used to evolve into respiratory insufficiency and death in half of the patients within five years after diagnosis. After infection by *P. aeruginosa*, however, only 8% would survive the 5-year time frame [15]. 

Kudoh [15] proved a decrease in symptoms, an improvement in pulmonary function, and a reduction in sputum volume and inflammatory factor levels, as well as a decrease in infection rates, resulting in a significant drop in disease-related mortality with the use of macrolides.

A retrospective study carried out by the Research Group of the Ministry of Health, Labor, and Welfare of Japan showed a pronounced improvement in survival rate after long-term use of macrolides at low doses. The 5-year survival rate before the treatment with macrolides was 63% in the 1970s, and 72% between 1980 and 1984. After the introduction of erythromycin in 1985, this rate increased to 92% (*P* < 0.0001) [16].

Since then, erythromycin has been the recommended choice of treatment upon diagnosis [15]. This therapeutic success is attributed to anti-inflammatory and immunemodulatory effects whose mechanisms have yet to be established.

The benefits of macrolides in the treatment of cystic fibrosis are plain to see [5, 17, 18]. Recent bibliographical review by Yousef and Jaffe proves this benefit through the increase in FEV1 and the decrease in pulmonary exacerbation [18]. The therapeutic success of azithromycin is attributed to the combination between the anti-inflammatory and antieffective effects of the drug. 

The use of macrolides has been investigated in asthma treatment [19–21]: a systematic review by Richeldi et al. [19] in 2008 involving 7 studies, with 416 participants, compared the use of macrolides in asthma to the use of a placebo. There was no statistically significant difference in the FEV1 values (forced expiratory volume in one second). Nevertheless, there was significant improvement in the symptoms scale and in the eosinophil count. The review concluded that the existing data are insufficient to support the systematized use of macrolides in asthma. 

### 1.1. Bronchiectasis

Bronchiectasis is a chronic pulmonary disease with a diverse etiology, characterized by recurrent respiratory infection and chronic inflammation, leading to the destruction of the airways and pulmonary parenchyma.

The treatment of bronchiectasis is currently based on the treatment of the underlying cause, on the prevention and control of respiratory infections, as well as on respiratory physiotherapy, and on surgical corrections, in more severe cases [22].

 Since 1997, several studies based on the knowledge acquired for the treatment of DPB have sought to learn more about the role of macrolides in bronchiectasis.

For the bibliographical research carried out until June 2011 with the use of the Medline database, we used the key words “macrolides” and “bronchiectasis”, seeking original papers and reviews. We found seven clinical studies that evaluated the action of macrolides on patients with bronchiectasis. Only two studies were randomized, placebo-controlled, and double blind (Table 1).

Koh et al. in a randomized, double-blind, placebo-controlled study with children at a mean age of 13.1 years (±2.6) with increased previous airway reactivity, using roxithromycin 4 mg/Kg twice a day for 12 weeks, evidenced a reduction in airway reactivity after the methacholine challenge test, in addition to an improvement in sputum purulence. However, it did not show any difference in sputum cellularity nor pulmonary function improvement when evaluating FEV1. The authors did not identify the roxithromycin mechanism of action in reducing the airway reactivity, but inferred anti-inflammatory or antimicrobial mechanisms. However, no conclusion was achieved, and the authors could not demonstrate the correlation between their findings and the clinical improvement [23]. 

Tsang et al. [24] evaluated the pulmonary capacity and sputum features in 21 adults with bronchiectasis after the use of erythromycin 500 mg twice a day for 8 weeks, in a double-blind, placebo-controlled study. They observed significant improvement in FVC (forced vital capacity) and FEV1 (*P* < 0.05) and a reduction in the 24-h sputum volume (*P* < 0.05). Nevertheless, there was no significant difference in the presence of microorganisms or in the presence of inflammatory products (IL-1*α*, IL-8, TNF*α*, leukotriene B4) in the sputum. Despite the small number of patients and the short duration of the study, there was an apparent reduction in pulmonary exacerbation frequency in the treated group in comparison to the placebo group. The mechanism of action of the drug is unknown, but it is unlikely to be bactericidal in view of the low dosage and poor tracheobronchial penetration. The authors believe that macrolides are a disease-modifying treatment in noncystic fibrosis bronchiectasis. Nevertheless, they underscore the importance of further studies in the field to determine dose response, appropriate duration of therapy, and criteria for patient selection.

Davies and Wilson in an attempt to reduce pulmonary exacerbation frequency in patients with noncystic fibrosis bronchiectasis, evaluated the pulmonary function and sputum features of 39 adults with a mean age of 51.9 years (±16.1), after treatment with azithromycin 500 mg once daily for 6 days, 250 mg once daily for 6 days, then 250 mg on Monday, Wednesday, and Friday for at least 4 months, and on average for 20 months. All patients had had at least four pulmonary exacerbations in the previous year. A total of 33 patients completed the 4 months of treatment and presented lower pulmonary exacerbation frequency with reduced need for oral (*P* < 0.001) and intravenous (*P* < 0.001) antibiotics when compared to their previous individual data. A pulmonary evaluation was carried out in 25 patients. Significant improvement was observed for all parameters (*P* = 0.01). Symptoms such as cough, fatigue, exercise tolerance, wheezing, and dyspnoea were evaluated using a 5-point scale. A significant improvement in all of the aspects evaluated was observed [25].

Cymbala et al. [26] investigated the pulmonary function and exacerbation frequency in 11 patients with bronchiectasis, after the use of azithromycin 500 mg twice weekly for 6 months. Patients were randomized in order to be given the usual treatment or the usual treatment plus azithromycin. Pulmonary function remained steady throughout the study. There was reduction in exacerbation incidence (*P* = 0.019) and reduction in sputum volume (*P* = 0.005) during the treatment and later at the control stage, which persists after the interruption of the treatment (*P* = 0.028), when compared to the individual data obtained 6 months prior to the intervention. Patients reported a boost in energy and an improved quality of life.

Yalcin et al., in a randomized and controlled study, evaluated 34 children aged between 7 and 18 (mean age 13.1 ± 2.7) with noncystic fibrosis bronchiectasis, after 3 months using clarithromycin at a dose of 15 mg/Kg/day. The control group was given supportive therapy only, whereas the study group received supportive therapy and clarithromycin. The presence of inflammatory mediators (IL8, TNF*α*, IL10) and the bronchoalveolar lavage (BAL) cellularity were evaluated, along with pulmonary function and sputum production. No important side effects were observed. The results evidenced a significant reduction in daily sputum production (*P* < 0.0001), improvement in FEF25–75% (*P* < 0.015) but not in the other spirometric parameters, in addition to a reduction in cellularity, the number of neutrophils, and in IL-8 levels, with increased macrophages ratios in BAL. No alteration was observed in the IL10 and TNF*α* levels, or in the rates of bacterial isolates throughout the study. The presence of microorganisms stimulates the production of TNF*α* by the bronchial epithelium. As there was no alteration to bacterial growth, the reduction in IL-8 levels and of neutrophils could be attributed to the anti-inflammatory effect of claritromicina. The isolated improvement in FEF25–75% was attributed to the removal of bronchial obstruction in the airways of small and medium caliber, probably due to reduction in inflammation at the bronchial wall, reduction in secretion to the bronchial lumen, and inhibition of cholinergic response in the airway smooth muscle. Despite the randomization, there was significant difference in the inflammation severity between the groups before the beginning of the study. Nonetheless, the fact that no control with placebo was used and no quantitative culture of BAL was performed may have impaired the validity of the study [27].

Anwar et al. described the use of azithromycin 250 mg three times a week in 56 adults (mean age 63 ± 12.9 years) with noncystic fibrosis bronchiectasis for at least 3 months, on an average of 9.1 months, in a prospective open study. This study compared the exacerbation frequency, the self-reported volume, and the microbiology of the sputum, as well as pulmonary function. The results obtained were compared to each patient's data obtained 6 months before the intervention. Pulmonary function was evaluated in 29 patients. The study evidenced a marked reduction in pulmonary exacerbation frequency (*P* < 0.001) and in the number of positive sputum cultures (*P* < 0.005); improvement in FEV1 (*P* < 0.002), without any improvement in FVC. All patients produced at least 1 tablespoon (>15 mL) of sputum daily, and at the end of the study, they no longer produced sputum [28].

Serisier and Martin in a prospective, open, noncontrolled study evaluated the exacerbation frequency and the use of Erythromycin in 21 patients aged 62.5 years (±11) on average, after the use of erythromycin 250 mg/day for 12 months. A comparison was made to each patient's individual data obtained prior to the 12-month time frame of the study. A reduction in the number of exacerbations (*P* < 0.0001) was detected, along with a reduction in the number of treatment days with oral (*P* < 0.0001) and intravenous (*P* < 0.0078) antimicrobial [29].

Recent British guidelines on nonfibrocystic bronchiectasis acknowledge the macrolides' possible immunemodulating effects and suggest that they may have a disease-modifying activity, while emphasizing the need for further studies on the subject before [22].

#### 1.1.1. Possible Mechanisms of Action

To date, there is a paucity of data exploring the mechanisms of action of macrolides in bronchiectasic patients as its clinical effectiveness has only been recently shown. Most of the data in this issue have been extrapolated from other chronic lung disease and animal or cell models or in vitro systems [18].

#### 1.1.2. Airway Condition in Chronic Inflammation

Diseases characterized by chronic airway inflammation, such as diffuse panbronchiolitis, asthma, chronic obstructive pulmonary disease (COPD), cystic fibrosis, and bronchiectasis, often bring with them mucus hypersecretion, bronchial hyperactivity, and chronic inflammation [30]. The process involves neutrophil inflammation with high levels of inflammatory cytokines IL1, IL8, and TNF*α* [28]. The inflammation, along with harmful bacteria-generated substances, may lead to notable tissue damage.

The authors suggest that the effect of macrolides on the improvement of chronic inflammatory conditions surpasses its antimicrobial effects, considering that in all the studies these drugs were administered in low doses, in which they would not present bactericidal or bacteriostatic effect [27]. 

It is believed that macrolides exert anti-inflammatory and immunomodulatory effects through the following mechanisms [31].

**(1). Effect on Airways Mucus**

Studies suggest that macrolides reduce expectoration by inhibiting the synthesis of mucus proteins, such as mucin, by modulating gene expression, and by inhibiting chloride channels in the epithelial cells. It is believed that they act in the mucociliary clearance and mucus viscosity, although these findings have yet to be explained [31].

**(2). Effects on Bacteria**

Gram-negative bacteria such as *Pseudomonas aeruginosa* produce an alginate biofilm with protective action against phagocytosis and antimicrobial agents. Infection starts with the adhesion of the host cells through adhesins. The tissue is then damaged by the toxins and enzymes produced by the bacteria. Macrolides reduce the biofilm formation, the production of molecules responsible for bacterial adhesion and mobility, and the secretion of cytotoxic compounds by these bacteria [31].

**(3). Immunomodulatory Effect**

Chronic inflammation is characterized by the recruitment of neutrophils with the release of lysosomal enzymes and the generation of reactive oxygen compounds, resulting in tissue damage. Macrolides reduce the quantity of neutrophils in the inflammation site by decreasing molecular adhesion (E-selectin, ICAM-1), integrins (CD11b/CD18), cytokines involved with chemotaxis (IL-8, IL-6, IL4, IL5), and TNF*α*.

Macrolides modulate phagocytosis indirectly reducing neutrophil survival by accelerating their apoptosis. They suppress the secretion of epithelial-derived neutrophil survival factors, as GM-CSF (granulocyte-macrophage colony-stimulating factor).

Initially, macrolides improve the host's defense through neutrophil stimulation, production of proinflammatory cytokines and mediators such as IL-1, IL-2, IL6, and GM-CSF, and production of nitric oxide in order to contain the infection [32]. However, continuous use attenuates chronic inflammation, through the suppression of inflammatory mediators (Il-8, eotaxin, TNF*α* and GM-CSF), thus limiting tissue damage [33].

## 2. Conclusions

Despite the small number of studies shedding light on the anti-inflammatory and immunomodulatory mechanisms of the macrolides, there is strong evidence providing support to the benefit of using this type of drug for the long term and in low doses to treat chronic inflammatory diseases, including noncystic fibrosis bronchiectasis.

The anti-inflammatory properties of macrolides are consolidated. The mechanisms of action, however, are still being investigated. 

Future studies are necessary to determine the benefits of macrolides in the many inflammatory diseases of the airways, as well as the ideal dosage and duration of the treatment, not to mention the impact of the development of bacterial resistance.

## Figures and Tables

**Table 1:** Macrolides in noncystic fibrosis bronchiectasis.

Study	Study design	*N*	Macrolide	Age (years)	End-point
Koh et al. [23]	Randomized, double-blind, placebo-controlled	25	Roxithromycin 4 mg/kg twice daily for 12 weeks	13.1 ± 2.6	(i) Reduction of sputum purulence (*P* < 0.005) (ii) Reduction in airway responsiveness after methacholine challenge (*P* < 0.01)

Tsang et al. [24]	Randomized, double-blind, placebo-controlled	21	Erythromycin 500 mg twice daily for 8 weeks	50 ± 15	(i) FEV1 and FVC improvement (*P* < 0.05) (ii) Reduction of 24-h sputum volume (*P* < 0.05) (iii) Reduction of the number of exacerbations

Davies and Wilson [25]	Prospective open-label	39	Azithromycin 250 mg, thrice weekly for 4 months	51.9 ± 16.1	(i) Reduction of clinical exacerbations with the use of oral and intravenous antibiotics (*P* < 0.001) (ii) Pulmonary function improvement (*P* < 0.01)

Cymbala et al. [26]	Randomized, open-label, crossover	11	Azithromycin 500 mg twice weekly for 6 months	—	(i) Reduction in pulmonary exacerbations (ii) Reduction of sputum volume

Yalcin et al. [27]	Randomized, controlled	34	Clarithromycin 15 mg/kg/day for 3 months	13.1 ± 2.7	(i) Reduction in bronchial inflammation (*P* < 0.02) (ii) Pulmonary function improvement (FEF25–75%) *P* < 0.015 (iii) Reduction of sputum volume (*P* < 0.0001)

Anwar et al. [28]	Prospective open-label	56	Azithromycin 250 mg, thrice weekly for at least 3 months	63 (±12.9)	(i) Reduction in pulmonary exacerbations (*P* < 0.001) (ii) Reduction of microorganisms rates in the sputum (*P* < 0.005) (iii) Reduction in the self-reported sputum volume (iv) FEV1 improvement (*P* < 0.002)

Serisier et al. [29]	Prospective open-label	21	Erythromycin 250 mg/day for 12 months	62.5 (±11)	(i) Reduction in pulmonary exacerbations (*P* < 0.0001) (ii) Reduction of antibiotics use (*P* < 0.0001)

FEV1: forced expiratory volume in one second; FEF25–75%: maximal midexpiratory flow; FVC: forced vital capacity.
